# Virtualized MME Design for IoT Support in 5G Systems

**DOI:** 10.3390/s16081338

**Published:** 2016-08-22

**Authors:** Pilar Andres-Maldonado, Pablo Ameigeiras, Jonathan Prados-Garzon, Juan Jose Ramos-Munoz, Juan Manuel Lopez-Soler

**Affiliations:** Department of Signal Theory, Telematics, and Communications, University of Granada, Granada 18071, Spain; pameigeiras@ugr.es (P.A.); jpg@ugr.es (J.P.-G.); jjramos@ugr.es (J.J.R.-M.); juanma@ugr.es (J.M.L.-S.)

**Keywords:** NFV, virtualization, 5G, LTE, M2M, IoT, traffic peaks

## Abstract

Cellular systems are recently being considered an option to provide support to the Internet of Things (IoT). To enable this support, the 3rd Generation Partnership Project (3GPP) has introduced new procedures specifically targeted for cellular IoT. With one of these procedures, the transmissions of small and infrequent data packets from/to the devices are encapsulated in signaling messages and sent through the control plane. However, these transmissions from/to a massive number of devices may imply a major increase of the processing load on the control plane entities of the network and in particular on the Mobility Management Entity (MME). In this paper, we propose two designs of an MME based on Network Function Virtualization (NFV) that aim at facilitating the IoT support. The first proposed design partially separates the processing resources dedicated to each traffic class. The second design includes traffic shaping to control the traffic of each class. We consider three classes: Mobile Broadband (MBB), low latency Machine to Machine communications (lM2M) and delay-tolerant M2M communications. Our proposals enable reducing the processing resources and, therefore, the cost. Additionally, results show that the proposed designs lessen the impact between classes, so they ease the compliance of the delay requirements of MBB and lM2M communications.

## 1. Introduction

The Internet of Things (IoT) is a term used for a set of technologies, systems and devices that enable connectivity to the Internet and that are based on the physical environment [1]. With a wide range of potential applications, IoT devices are rapidly spreading, and forecasts are predicting a huge growth of these devices over the next few years [2].

Recently, cellular systems are being considered as an option to provide connectivity to IoT devices due to their ubiquitous presence, widespread coverage, reliability and support for mobility [3]. Within the cellular context, the IoT connectivity solution is referred to as Machine-to-Machine (M2M). However, cellular systems have been designed for Human to Human communications (H2H) or Mobile Broadband Access (MBB), and consequently, the widespread provision of M2M services with these systems entails significant technical challenges [4]. Some of these challenges are: the scalability issues raised by the huge number of expected devices; the time-varying traffic characteristics of M2M applications, which are very different from MBB or H2H; and the very heterogeneous QoS demands in terms of bandwidth, latency and reliability.

The 3rd Generation Partnership Project (3GPP) has included enhancements in Long-Term Evolution (LTE) networks for the deployment of IoT. One of them is data transport in Control Plane Cellular IoT Evolved packet system Optimization (CPCEO) [5]. This is a set of transmission procedures specifically designed for small and infrequent data transmissions from/to M2M devices. CPCEO procedures employ Non-Access Stratum (NAS) messages to transfer data packets from/to the device, instead of the establishment and utilization of data plane bearers.

The adoption of the CPCEO procedures can mitigate relevant issues caused by small and infrequent data packet transmissions by a huge number of M2M connected devices. CPCEO procedures reduce the signaling explosion generated by the establishment and release of data bearers. This reduction affects the core and especially the radio interface due to the limited radio resources. However, CPCEO procedures imply a major increase of the processing load on the control plane of the Evolved Packet Core (EPC) and, in particular, on the Mobility Management Entity (MME). In addition, the current exposition of the EPC entities to the signaling in LTE [6], combined with the fixed capacity of current core LTE hardware-based infrastructure, can limit the scalability of the CPCEO solution.

Currently, major efforts are being made to research and develop new technologies for future 5G systems [7]. Network Function Virtualization (NFV) is one of the promising new technologies for 5G. NFV provides a novel framework to deploy network services onto virtualized servers. The use of the NFV paradigm to virtualize the EPC entities and, in particular, the MME could facilitate the wide deployment of M2M communications in 5G. It improves the scalability and flexibility of the network compared to hardware-based entities. This benefit is crucial for the foreseen signaling explosion generated by M2M connected devices. Due to the promising benefits of NFV, there are works that have tackled the architectural and implementation issues of applying this virtualization paradigm to the LTE EPC; see, for example, [8,9,10,11]. However, these proposals do not specifically consider support for M2M traffic.

In this paper, we propose two designs for a virtualized MME that specifically aim at facilitating the IoT support in 5G systems. The first proposed design partially separates the processing resources dedicated to different traffic classes; while the second design includes traffic shaping to control the traffic of each class. In our system, we have considered MBB, low latency M2M and delay-tolerant M2M traffic classes. Our proposed schemes adjust the processing resources considering the traffic classes to serve and their QoS requirements.

We have analyzed the running costs of the resources needed and the delay performance. We have compared our proposed schemes to two other schemes: (i) a baseline virtualized MME design, which does not apply any specific traffic treatment per class; (ii) an overdimensioned virtualized MME. For the cost analysis, we have considered a theoretical model to dimension the required resources, and the data center setup and billing model of the Amazon Elastic Compute Cloud (EC2). For the delay performance evaluation, we have simulated each scheme.

The results show that our schemes provide much lower costs than the overdimensioned one. Furthermore, they provide similar delay performance for MBB and low latency M2M communications. The obtained delay satisfies the exigent requirements of MBB and low latency M2M communications. However, our proposed schemes apply a specific delay requirement per traffic class. This allows the saving of processing resources for delay-tolerant M2M traffic.

The remainder of this paper is organized as follows. Section 2 gives an introduction to the main topics on which this work is based and poses the addressed problem. Section 3 introduces a detailed description of the system model and assumptions made. The adopted traffic models are explained in Section 4. In Section 5, we explain the considered schemes to study, including our proposed schemes. Section 6 presents the queue model used in the dimensioning of the virtualized MME. Section 7 analyzes the results. Finally, the conclusion is in Section 8.

## 2. Background

### 2.1. Internet of Things

The convergence of the digital and the physical world provided by the Internet of Things (IoT) allows the interaction between the environment and the devices connected to the network that collect information. Under this umbrella, many applications can be envisaged, which have led to several markets (verticals) for IoT. In IoT applications, machines (or devices) connected to the network can communicate among them, or with the application server, without human interaction. This is known as Machine-to-Machine (M2M) communications.

IoT applications are designed for specific verticals, such as industry (e.g., monitoring industrial plants, boarding operation), energy (e.g., inventory, waste collection), automotive (e.g., bike sharing, parking system), healthcare (e.g., diagnostics, mobile assistance) or media and entertainment (e.g., payment systems, comfortable living) [12]. These verticals have different requirements that determine IoT applications’ design, as for example, the particular connectivity needs, device energy consumption, capabilities, security or traffic characteristics.

Despite there being many possible requirements for IoT applications, M2M communications used in IoT are being classified by organizations, such as the 3GPP or the Mobile and wireless communications Enablers for the Twenty-twenty Information Society (METIS), in two big trends [13], based on their Quality of Service (QoS) requirements:

- Massive M2M (mM2M): characterized by low cost/energy devices, small data volumes and a massive number of devices connected. Among others, relevant examples are smart metering, fleet tracking or building automation.
- Low latency M2M (lM2M): characterized by strict requirements, such as ultra reliability, low latency and high availability. The distinct requirements depend on the specific application. For instance, the end-to-end latency can reach down to a few milliseconds or even lower [14]. To achieve that, low latency services could be served by different core entities in the network. For simplicity, we assume that the lM2M devices considered in this work can be handled by the MME. Industrial monitorization, security or healthcare, and tactic Internet are examples of this trend.

**Figure 1:**
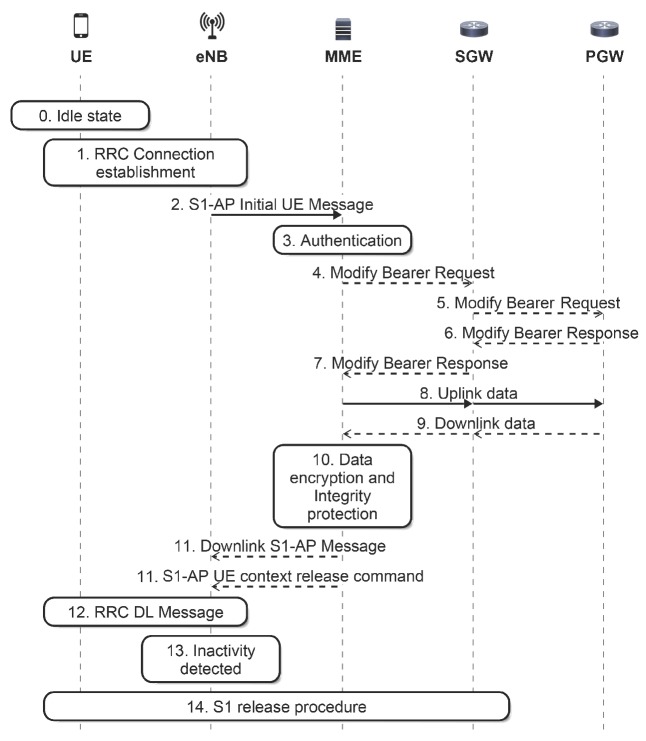
Mobile-originated data transport in the NAS packet data unit [5].

For most of M2M communications used in IoT applications, small and infrequent data transmissions are a common traffic characteristic, as mentioned in [15]. Nevertheless, this traffic characteristic is handled inefficiently by current mobile networks, such as LTE. This is mainly due to the prior resource reservation required before the transmission [16]. To solve that, the 3GPP has included LTE enhancements for M2M in its specifications, recently integrated into the context of Cellular IoT (CIoT). For example, the 3GPP has introduced in [5] new procedures to reduce signaling to transfer data packets from or to the device, named data transport in Control Plane CIoT Evolved packet system Optimization (CPCEO). To this end, the procedures use Non-Access Stratum (NAS) transport capabilities. In LTE, NAS is used to transfer non-radio signaling between the User Equipment (UE) and the MME. In the transmission of uplink data, with this new procedure, the data packet is sent as NAS signaling by the UE to the MME after Radio Resource Control (RRC) establishment. Next, the MME carries out the security check, and then, it forwards the data packet to the Serving Gateway (SGW) (see Figure 1). The CPCEO procedures avoid the establishment and release of data plane bearers for small and infrequent packets from M2M devices, but the MME has to process these packets from the data plane transmitted as NAS signaling.

In the LTE Evolved Packet Core (EPC) architecture, the MME deals with the control plane. That is, this entity is the main signaling node in the EPC. Its main functions are: NAS signaling, user authentication, mobility management (e.g., paging, user tracking) and bearer management. For more information about the MME entity, see [5]. Therefore, the main drawback of these new procedures for cellular IoT is the increased processing capacity required by the MME [17].

In addition to this transfer optimization, a new narrowband radio technology for IoT was developed in Release 13, called the Narrowband Internet of Things (NB-IoT). The objective of NB-IoT is to reduce complexity, increase coverage, achieve long battery lifetime and support a massive number of devices. To achieve these objectives, NB-IoT has low data rates, a 164-dB maximum coupling loss target for standalone mode operation and limited mobility support, and it allows only the half-duplex frequency-division duplexing operation. NB-IoT requires a reduced bandwidth of 180 kHz for both downlink and uplink and further protocol optimizations [18]. For UEs that support the NB-IoT, the 3GPP agreed that the control plane CIoT Evolved Packet System optimization feature explained above will be mandatory [5]. For more information about NB-IoT, see [19].

### 2.2. Virtualization

NFV is one of the enabling technologies of 5G systems, because it will allow network operators to cope with challenges, such as the traffic increase and IoT support while reducing CAPEX and OPEX. With NFV, network functionalities are implemented with Commercial Off-The-Shelf (COTS) hardware, which is much less expensive than the very specialized hardware used in 4G networks. In this way, operators can create, scale and deploy network components whenever they are needed, according to the particular real-time traffic conditions. These benefits are relevant for the deployment of IoT in cellular networks. NFV could facilitate the adaptation of the network entities to the new demand. With NFV, the infrastructure services can be programmed, instead of re-architecting the infrastructure of the network [20].

In [9], the authors discuss the design of a virtualized EPC for implementation in a cloud computing environment and its offering “as a Service” (EPCaaS). They present and analyze a number of different implementation options for the virtualized EPC. Focusing on the 1:N mapping option, each entity of the EPC is decomposed into three logical components: a Front End (FE), Service Logic (SL) and State Database (SDB). The FE acts as a communication interface with other entities of the network, for example the FE terminates Stream Control Transmission Protocol (SCTP) sessions between eNodeB and MME [21] and balances the load among several SLs. SLs implement the processing. The SDB stores the user session state, making the SLs stateless. This design follows the multi-tiered web services paradigm for cloud-based applications. Each of the three logical components is a tier of the multi-tier architecture. SLs are stateless, and therefore, they can scale out/in without impacting externally-connected peers and independently from other SLs. The scaling of SDB and FE, which are stateful components, is possible [22], although it requires a careful design.

In multi-tiered web services, dedicated hosting refers to the case where a server is allocated to at most one application at any given time [23]. On the contrary, in shared hosting, multiple small applications share each server. Dedicated hosting is used for running large clustered applications where server sharing is unfeasible due to the workload demand imposed on each individual application.

Dimensioning is the classic process to determine the amount of resources required to process a set of requests with a specific Quality of Service (QoS) target. However, dimensioning is a static process, whereas the traffic in mobile networks strongly fluctuates. However, dynamic provisioning of resources according to the fluctuations in the request rate is feasible thanks to the virtualization technologies and the wide adoption of cloud computing system infrastructures [24]. As in the case of Internet workloads, mobile network traffic exhibits long-term variations, such as time-of-day or seasonal effects, as well as short-term fluctuations caused by unexpected events. Therefore, it is reasonable to consider applying dynamic provisioning techniques of multi-tiered web services for virtualized EPC entities.

In [22], the authors study the provision resources in multi-tier applications and propose a strategy to avoid bottleneck shifting. They propose to break down the end-to-end response time $\bar{D}$ into per-tier response times ${\bar{D}}_{1}$, ${\bar{D}}_{2}$,... ${\bar{D}}_{j}$, such that:

(1) $$\sum_{j=1}^{j}{\bar{D}}_{j}=\bar{D}.$$

Then, they propose to determine how many servers to allocate to each tier separately, such that tier *j* processes the requests with a service response time equal to ${\bar{D}}_{j}$. For this capacity prediction, an estimate of the peak session rate is used. In Section 5, we will use this strategy in the estimation of resources for the virtualized MME entity.

We will further assume that the virtualized EPC entities are deployed using a cloud provider service, such as Amazon’s Elastic Computing Cloud (EC2), in which the computing resources can be dynamically scaled in or out on demand and with a pay-as-you-go price scheme. For example, Amazon EC2 charges a per-hour price for the service use [25]. Additionally, a relevant aspect in cloud services such as EC2 is that the time required to obtain and boot a new server instance can be in the range of tens of seconds to minutes [26].

### 2.3. Characteristics of M2M Traffic in Cellular Networks

The penetration of connected machines in the near future is expected to outnumber human connections, reaching values up to one order of magnitude above [27]. This number and the traffic characteristics of M2M devices make difficult their wide deployment in current cellular networks, such as LTE [28].

**Figure 2:**
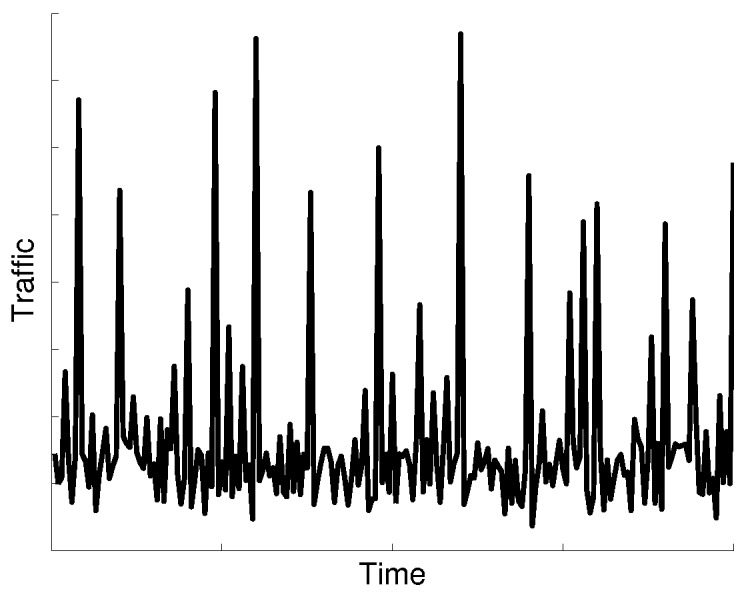
Example of M2M traffic peaks.

M2M communications have different characteristics compared to conventional human-based communications. M2M average traffic volume is small, about two orders of magnitude lower than MBB traffic volume. The M2M ratio of uplink traffic volume is higher than downlink traffic volume. Furthermore, synchronized communication from a large number of devices causes traffic peaks, e.g., in periods of 1 h, 30 min or 15 min, as shown in the time series analysis of M2M traffic [29]. Besides report transmission synchronization events, there will be alarms caused by unpredictable events. Together, all of these events could imply peaks of signaling load two- or four-times higher than the average load [30]; see Figure 2.

From the network point of view, the scalability of the control procedures triggered by these transmissions is one of the most important challenges for M2M support. As with the CPCEO, the MME is to convey these data packets; it has to be designed to be able to deal with this traffic increase and to avoid any associated congestion. Dynamic provisioning of processing resources is of little use for the vMME to carry the traffic caused by these traffic peaks. First, the billing models of cloud providers (such as Amazon EC2) typically charge per hour, and traffic peaks usually occur at shorter time intervals. Second, there will be M2M traffic peaks caused by alarm events that cannot be predicted, and therefore, they would cause congestion during the tens of seconds or minutes needed to boot new server instances. For these reasons, we will assume that the amount of resources required to serve these traffic peaks has to be dimensioned in advance.

## 3. System Model

**Figure 3:**
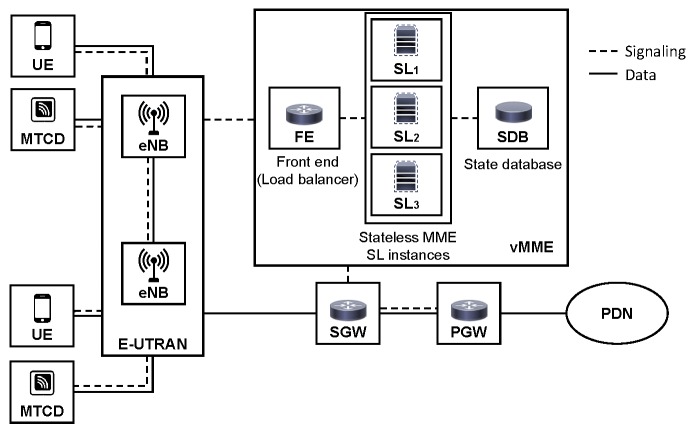
Overall system model.

In this work, we assume an access cellular network architecture based on LTE, which provides service to Users Equipment (UE) and Machine-Type Communications Devices (MTCDs). Although this architecture assumes the entities defined in LTE/EPC, it could also be extended to other cellular architectures. The overall system model is depicted in Figure 3.

The main entities are explained next. Additionally, the notation and main definitions used in this work are summarized in Table 1.

### 3.1. The User Equipment

UEs are the terminals that allow each human user to connect to the network via the eNodeB base stations. The UEs run the users’ applications, which generate or consume network traffic (see the traffic model in Section 4). If a UE is in the idle state and it has data to transmit, it carries out a Service Request (SR) procedure to establish a bearer, which will convey the data packets. We assume that UEs move following a fluid-flow mobility model. When a UE crosses the border between two cells, it triggers an X2-based Handover (HO) procedure.

### 3.2. MTC Devices

We assume that MTCDs are placed in fixed locations, and they send small data packets (reports) infrequently to centralized servers. Following the 3GPP and METIS guidelines (see Section 2.1), we consider two types of MTCDs, mM2M and lM2M devices. We assume mM2M devices run delay-tolerant M2M applications, which can tolerate seconds of delay [31], such as smart metering or agriculture applications. We also assume that lM2M devices run strict M2M applications, such as industrial applications or healthcare, which are characterized by low latency requirements among other demands. We also assume a large number of MTCDs connected to the network, which will cause traffic peaks due to M2M synchronization or alarm events. For simplicity, we assume there is no coordination among mM2M and lM2M events.

As the M2M ratio of uplink traffic volume is higher than downlink (see [29]), we only consider uplink M2M traffic. To transmit a report, we assume that an M2M device triggers the Mobile-Originated data transport signaling procedure, defined in the Control Plane CIoT Evolved packet system Optimization (MO-CPCEO) [5]. Regarding the MO-CPCEO procedure, we assume that the following control messages do not take place (see Figure 1):

- Messages 4 to 7 are not used, since there is a connection established between MME and SGW, and there is no important change to inform the Packet data network Gateway (PGW);
- Messages 9 to 13 are not used as, no downlink data are expected by the MTCD.

Then, the MME has to process one control message for each MO-CPCEO procedure.

### 3.3. eNodeB Stations

eNodeB (eNB) stations receive signaling messages from the UEs and forward them to the virtualized MME. Each eNB keeps a user inactivity timer, which has an expiration time $T_{I}$, for each attached UE within its coverage area. Using this timer, the eNB detects the users’ inactivity (i.e., a user does not perform any data transmission over a period of length $T_{I}$). If the timer expires, the eNB triggers a Service Release (SRR) procedure to release the bearer [5].

### 3.4. The Virtualized Mobility Management Entity

The virtualized MME (vMME) is the main control plane entity of the network. It maintains the mobility state of the UE and is responsible for the bearers’ management. For simplicity reasons, we assume that the vMME is collocated with the SGW and the PGW at a centralized data center of the network.

We consider that the MME is virtualized following the NFV paradigm, and in particular, we adopt the 1:N mapping architectural option. Thus, following the implementation in [9,21], the vMME is split into three tiers: FE, MME SL and SDB. We further assume that when an MME SL instance finishes processing a control plane message, it saves the transaction state into the SDB. When a subsequent request arrives at an MME SL instance, it first gathers the transaction state from the database from which to continue. This differs from the vMME implementation in [21], and it allows fully stateless MME SLs. Different messages of the same procedure for the same user can be processed by different MME SL instances. Therefore, the number of MME SL instances, denoted as $m_{SL}$, can grow without affecting in-session users.

As in [22], we assume that all tiers can be replicated within limits. When the processing capacity assigned to the vMME cannot withstand the current load, a new MME SL instance must be instantiated, and a new processor is added to the processing resources pool.

For simplicity, we will assume that every processor in the data center facility provides the same computational power, and the SDB follows a shared-everything architecture, which eases the scale-out or scale-in. Then, the scaling of the SDB can be done on-demand, but with certain constraints [22]. These constraints are due to the shared resources by multiple processors, which may result in choking off the bandwidth for simultaneous memory access or difficult synchronization mechanisms to maintain a shared consistent state. Furthermore, we will assume each tier has enough memory resources to store an unlimited amount of requests that arrive at the tier, while they are waiting to be processed.

## 4. Traffic Models

We assume three classes of traffic: Mobile Broadband traffic (MBB), massive M2M (mM2M) and low latency M2M (lM2M) traffic. In the next subsections, we explain each one.

### 4.1. Mobile Broadband Traffic Model

We adopt the compound data traffic and user behavior model proposed in [32] for MBB UEs. This traffic model considers three types of applications, namely:

1. Web browsing.
2. HTTP progressive video (e.g., YouTube).
3. Video calling (e.g., Skype service).

which are redesigned to generate the data rates predicted for future mobile networks in the METIS project [33]. Then, the mean data rates per user used are higher than the current demand. For web browsing, the mean data rate per user depends on the number of web pages visited, the main object size of each web and the number of embedded objects and their sizes. For HTTP progressive video, the mean data rate per user depends on the number of downloaded video clips, the size of each video and the video encoding rate. While for video calling, the mean data rate per user depends on the constant bit rate of the call. Consequently, from [32], the mean data rates per user obtained are 233.28 kbps, 5.25 Mbps and 142.07 kbps for web browsing, HTTP progressive video and video calling, respectively.

Most of the signaling workload generated by UEs depends on their activity and their traffic characteristics [32]. Here, we only consider those LTE control procedures that generate the most signaling load on the MME [10]. In particular, we consider Service Request (SR), Service Release (SRR) and X2-based Handover (HO) procedures. According to [32], for each of these procedures, the MME respectively will process 3, 3 and 2 control messages.

Let $N_{MBB}$ be the number of MBB UEs and ${\bar{\lambda }}_{SR}$, ${\bar{\lambda }}_{SRR}$ and ${\bar{\lambda }}_{HO}$ be the mean generation rate per UE for the SR, SRR and HO control procedures, respectively. Let ${\bar{\lambda }}_{MBB}$ be defined as the mean arrival rate of control messages processed by the MME that are generated by MBB UEs. Then, it can be computed as:

(2) $${\bar{\lambda }}_{MBB}=N_{MBB}\cdot \left(3\cdot {\bar{\lambda }}_{SR}+3\cdot {\bar{\lambda }}_{SRR}+2\cdot {\bar{\lambda }}_{HO}\right)$$

### 4.2. Machine to Machine Traffic Model

We consider two types of M2M devices: massive M2M (mM2M) devices and low latency M2M (lM2M) devices. Let $p_{h}$, where $h\in \left\{mM2M,lM2M\right\}$, denote the percentage of each type of M2M devices, and let *r* be the ratio of M2M devices per MBB UE. Then, the number of M2M devices of each type, $N_{h}$, is given by:

(3) $$N_{h}=N_{MBB}\cdot r\cdot p_{h}$$

**Figure 4:**
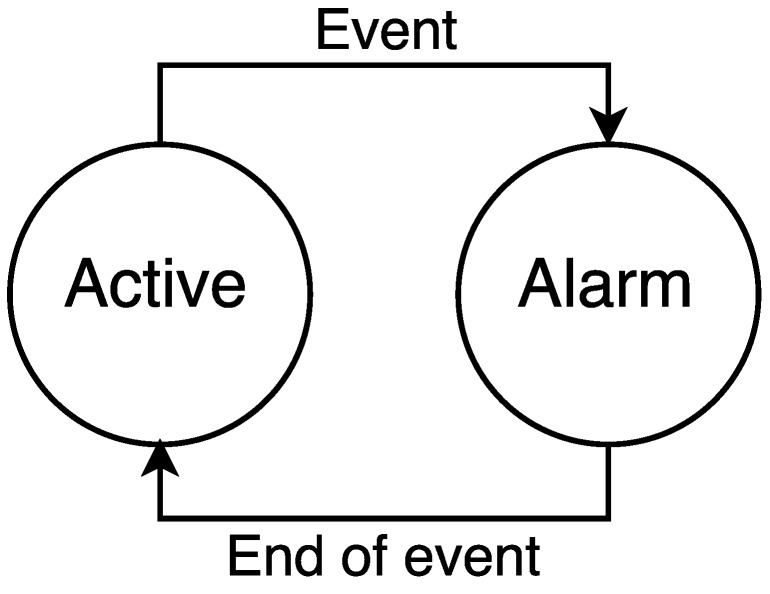
M2M device state transition diagram.

For both types of M2M devices, we assume a traffic model based on the transmission of reports. Particularly, the traffic model of any M2M device has two possible states: *active* and *alarm* (see Figure 4). M2M devices generate small data packets following a Poisson process in both states. In the *active* state, packets are generated infrequently, with a mean rate ${\bar{\lambda }}_{active}$. When an event happens, a percentage of massive or low latency M2M devices, denoted by $a_{h}$ where $h\in \left\{mM2M,lM2M\right\}$, change to the *alarm* state. During the *alarm* state, M2M devices generate packets more frequently, with a mean rate denoted as ${\bar{\lambda }}_{alarm}$. We assume that the event has a fixed time duration $t_{e}$, and the probability that in a certain instant of time an M2M device is in the *alarm* state is denoted as $p_{h}^{e}$, where $h\in \left\{mM2M,lM2M\right\}$. After the event, all devices in the *alarm* state return to the *active* state.

Massive M2M device events occur at certain instants in time caused by a large number of mM2M devices synchronizing their report transmissions (see Section 2.3). Some of these events are periodic, whereas others are not. For simplicity, in our model, we assume that mM2M synchronization events take place at periodic instants of time. On the other hand, lM2M events are caused by alarm situations; that is, they are unpredictable. Consequently, in our model, lM2M events occur at random instants in time.

Furthermore, we assume that the percentage of M2M devices participating in a synchronization event, $a_{h}$, is a discrete random variable. By appropriately choosing ${\bar{a}}_{h}$, ${\bar{\lambda }}_{active}$ and ${\bar{\lambda }}_{alarm}$, we can model the traffic peaks (as explained in Section 2.3).

**Figure 5:**
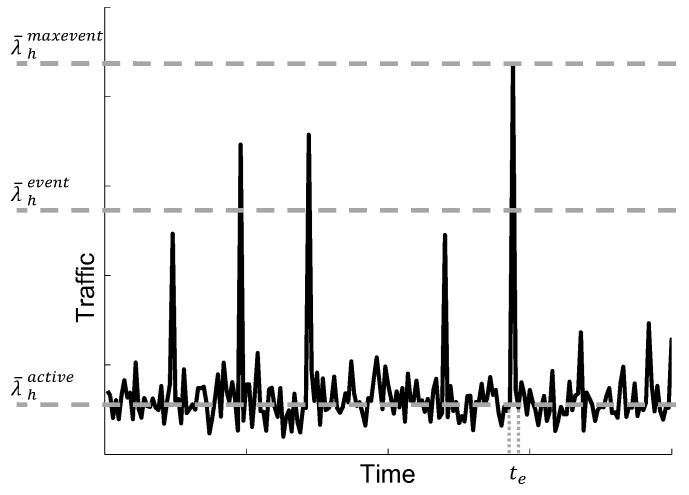
Parameters for the M2M traffic model.

Let us analyze the mean generation rate of the MO-CPCEO procedure per M2M class. As there is one control message per MO-CPCEO procedure (see Section 3.2), the mean generation rate of the MO-CPCEO procedure also defines the mean arrival rate of the MO-CPCEO control messages per M2M class to be processed by the MME. Let us denote this mean arrival rate as ${\bar{\lambda }}_{h}^{v}$. The parameter *v* defines the time interval used for averaging the arrival rate. Particularly, *v* can take three possible values $\left\{active,event,maxevent\right\}$, which corresponds to the three following different traffic situations (see Figure 5):

- Active: intervals of time in which all M2M devices are in the *active* state; that is, in the absence of events.
- Event: intervals of time in which an event takes place; that is, intervals of time where $N_{h}\times a_{h}$ devices are in the *alarm* state. The averaging is carried out over all possible events, each with a given $a_{h}$.
- Maxevent: time interval in which the largest event takes place; that is, the interval of time where $N_{h}\times max\left(a_{h}\right)$ devices are in the *alarm* state.

Then, for each averaging time interval, ${\bar{\lambda }}_{h}^{v}$ can be calculated as:

(4) $$\begin{array}{cc} \text{Mean}\;\text{rate}\;\text{in}\mathrm{active}\; & {\bar{\lambda }}_{h}^{active}=N_{h}\cdot {\bar{\lambda }}_{active}\\ \text{Mean}\;\text{rate}\;\text{in}\mathrm{event}\; & {\bar{\lambda }}_{h}^{event}=N_{h}\cdot \left[\left(1-{\bar{a}}_{h}\right)\cdot {\bar{\lambda }}_{active}+{\bar{a}}_{h}\cdot {\bar{\lambda }}_{alarm}\right]\\ \text{Mean}\;\text{rate}\;\text{in}\mathrm{maxevent}\; & {\bar{\lambda }}_{h}^{maxevent}=N_{h}\cdot \left[\left(1-max(a_{h})\right)\cdot {\bar{\lambda }}_{active}+max\left(a_{h}\right)\cdot {\bar{\lambda }}_{alarm}\right] \end{array}$$

Let ${\bar{\lambda }}_{h}$, with $h\in \left\{mM2M,lM2M\right\}$, be the mean arrival rate of MO-CPCEO control messages to the vMME per M2M class. For ${\bar{\lambda }}_{h}$ estimation, the entire period of time is considered. Then, it follows that:

(5) $${\bar{\lambda }}_{h}=\left(1-p_{h}^{e}\right)\cdot {\bar{\lambda }}_{h}^{active}+p_{h}^{e}\cdot {\bar{\lambda }}_{h}^{event}$$

## 5. Virtualized MME Design for M2M

This section describes the main points relevant to the virtualized MME (vMME) design adopted. Firstly, we introduce a design parameter named the *target arrival rate per tier* . This parameter defines the arrival rate used in the dimensioning of the vMME resources. Secondly, we explain two baseline schemes assumed in this work, named the Baseline Scheme (BS) and the Overdimensioned Scheme (OS). These baseline schemes highlight the impact of the overdimensioning of resources due to the inclusion of M2M events. Finally, the last subsection explains the two proposed schemes, called the Traffic separated Scheme (TS) and the traffic Shaper Scheme (SS). These proposed schemes include new mechanisms to optimize the dimensioning of the vMME, while at the same time they satisfy to the *target arrival rate per tier* defined.

### 5.1. Target Arrival Rate Design

The dimensioning of the schemes is done at each tier of the vMME separately, based on [22]. To dimension the resources needed at each tier of the vMME model in advance, we define a design parameter name the *target arrival rate per tier*. The target arrival rate is the maximum arrival rate of control messages for which the vMME has to satisfy a mean response time budget (${\bar{D}}_{j}$) for tier *j*. That is, the vMME is required to have sufficient processing capacity at tier *j* to satisfy ${\bar{D}}_{j}$ with an arrival rate up to the target.

On the one hand, for MBB communications, we set the target arrival rate, $\lambda_{MBB}^{t}$, equal to the mean arrival rate of MBB control messages (see Section 4.1), then $\lambda_{MBB}^{t}={\bar{\lambda }}_{MBB}$. On the other hand, for M2M communications, the target arrival rate $\lambda_{h}^{t}$, with $h\in \left\{mM2M,lM2M\right\}$, depends on whether we choose the vMME to satisfy ${\bar{D}}_{j}$ at each tier *j* during the traffic peaks caused by M2M events. We consider the following three approaches to serve the traffic peaks:

- *Peak* : denoted by $\lambda_{h}^{peak}$, we set the target arrival rate equal to the mean arrival rate during the largest event, ${\bar{\lambda }}_{h}^{maxevent}$. With this setting, each tier *j* of the vMME has to satisfy ${\bar{D}}_{j}$ for all traffic peaks, including the largest one. Therefore, it follows that:
  (6) $$\lambda_{h}^{t}=\lambda_{h}^{peak}={\bar{\lambda }}_{h}^{maxevent}$$
- *Intense smoothing of peaks* : denoted by $\lambda_{h}^{intsmooth}$, we set the target arrival rate equal to a weighted sum of the mean arrival rates in absence of an event and during an event (see Equation (4)). Therefore,
  (7) $$\lambda_{h}^{t}=\lambda_{h}^{intsmooth}=\left(1-w_{h}\right)\cdot {\bar{\lambda }}_{h}^{active}+w_{h}\cdot {\bar{\lambda }}_{h}^{event}$$
  The parameter $w_{h}$, with $h\in \left\{mM2M,lM2M\right\}$, defines the weighting factor of both M2M arrival rates. We select $w_{h}$ such that $\lambda_{h}^{intsmooth}$ is slightly higher than ${\bar{\lambda }}_{h}$ (see Equation (5)). With this setting, each tier *j* of the vMME is not able to satisfy ${\bar{D}}_{j}$ during the traffic peaks. Part of the signaling messages arriving during a traffic peak will be served after the end of the event.
- *Moderate smoothing of peaks*: denoted by $\lambda_{h}^{smoothpeak}$, we set the target arrival rate equal to a weighted sum of the mean arrival rates in absence of an event and during the largest event (see Equation (4)). Therefore, it follows that:
  (8) $$\lambda_{h}^{t}=\lambda_{h}^{smoothpeak}=\left(1-w_{h}\right)\cdot {\bar{\lambda }}_{h}^{active}+w_{h}\cdot {\bar{\lambda }}_{h}^{maxevent}$$
  With this setting, each tier *j* of the vMME is also not able to satisfy ${\bar{D}}_{j}$ during the traffic peaks. However, $\lambda_{h}^{smoothpeak}>\lambda_{h}^{intsmooth}$; therefore, the signaling messages arriving during a traffic peak will be served faster than in the previous case.

### 5.2. Baseline Schemes

#### 5.2.1. Baseline Scheme

**Figure 6:**
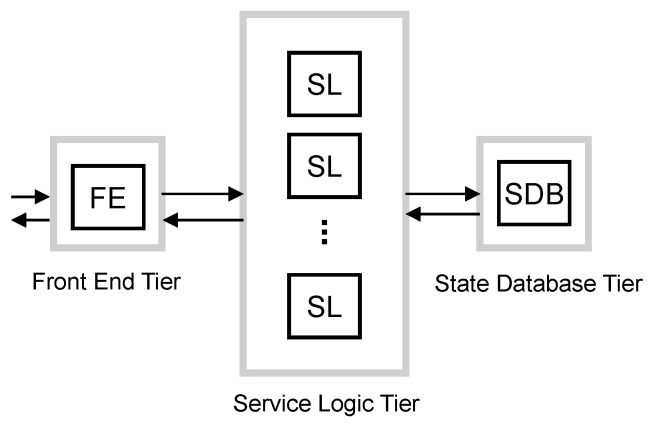
Baseline scheme block diagram.

This scheme is based on the system model presented in Section 3 and also shown in Figure 6. BS is composed of the aforementioned three tiers:

- FE: Front End,
- SL: Service Logic and
- SDB: State Database tier.

In this scheme, all resources are shared by all traffic classes, and all tiers have to convey the same target arrival rate.

For the baseline scheme, we set the target arrival rate such that each tier is able to serve the mean arrival rate from MBB UEs, mM2M devices and lM2M devices. Hence, with this scheme, each tier *j* is not able to satisfy ${\bar{D}}_{j}$ during the M2M traffic peaks. For them, we apply the intense smoothing approach mentioned above. Hence, the vMME will be overloaded for a period of time during and after traffic peaks.

Let $\lambda_{FE}$, $\lambda_{SL}$ and $\lambda_{SDB}$ respectively denote the target arrival rate of control messages in the FE tier, the SL tier and the SDB tier. In BS, we calculate them as:

(9) $$\lambda_{FE}=\lambda_{SL}=\lambda_{SDB}=\lambda_{MBB}^{t}+\lambda_{mM2M}^{t}+\lambda_{lM2M}^{t}={\bar{\lambda }}_{MBB}+\lambda_{mM2M}^{intsmooth}+\lambda_{lM2M}^{intsmooth}$$

Note that this design requires enough memory resources to store all packets while they are queued, as assumed in Section 3.

#### 5.2.2. Overdimensioned Scheme

The second baseline scheme considered is the same as the baseline scheme, but in this case, it is overdimensioned to satisfy ${\bar{D}}_{j}$ at each tier of the vMME at all times, including during the traffic peaks caused by M2M devices.

Hence, this scheme is designed with a target arrival rate equal to the sum of the mean arrival rate for MBB and the mean arrival rate during the largest event of mM2M and lM2M devices. Since we assume there is no coordination of mM2M and lM2M events (see Section 3.2), the resources do not need to be designed for simultaneous mM2M and lM2M events. Peaks from mM2M devices are expected to have a bigger impact on the vMME because of the massive number of mM2M devices. Then, we focus the arrival rate on mM2M peaks, using the mean arrival rate during the largest mM2M traffic peak, ${\bar{\lambda }}_{mM2M}^{maxevent}$. Therefore, the target arrival rate used in this scheme, which is the same for all tiers, can be computed as:

(10) $$\lambda_{FE}=\lambda_{SL}=\lambda_{SDB}={\bar{\lambda }}_{MBB}+\lambda_{mM2M}^{peak}+\lambda_{lM2M}^{intsmooth}$$

### 5.3. Proposed Schemes

#### 5.3.1. Traffic Separated Scheme

To mitigate the overdimensioning caused by the synchronization events of massive M2M devices, the first proposed scheme separates the processing of the traffic classes. To do that, we propose to use dedicated resources and dimension them separately for each traffic class. However, it is challenging to apply such separation at all tiers.

Regarding the FE tier, the vMME has to be seen as a single entity by the remaining elements of the 3GPP architecture. Additionally, the FE has to classify the traffic into the defined traffic classes. For these reasons, we do not consider the option of dividing the FE tier per traffic class. In addition, we also do not consider the option of dividing the SDB tier per traffic class. The rationale is that the SDB is considerably more expensive than the other elements of the design [34], and therefore, such a division per traffic class would yield a costly and, therefore, unattractive design.

**Figure 7:**
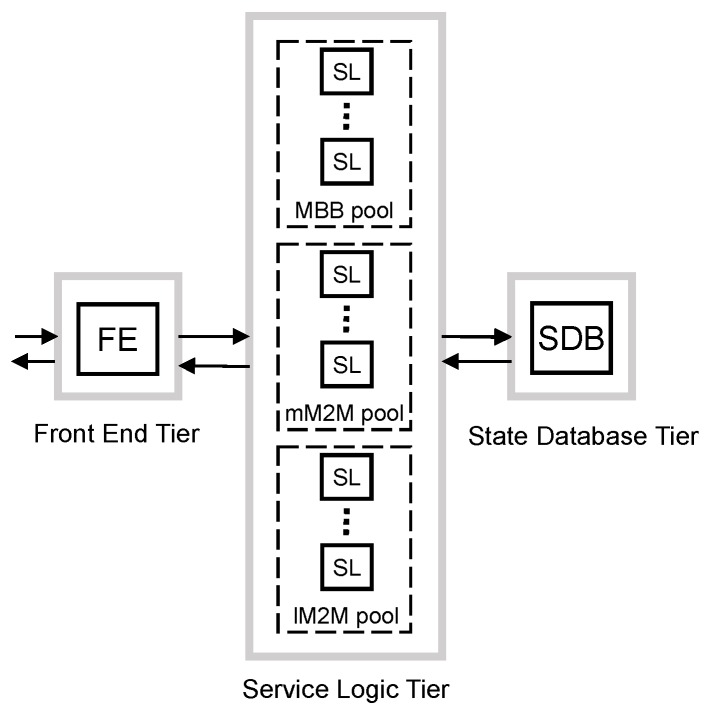
Traffic separated scheme block diagram.

Consequently, we apply the separation of dedicated resources for each traffic class only at the SL tier (see Figure 7). The benefit of this scheme is that the target arrival rate of each pool of SL can be optimized using the main traffic characteristics of the traffic served. Additionally, the mean response time budget ${\bar{D}}_{j}$ at the SL tier can be set differently for each traffic class.

This scheme, unlike previous ones, has a different target arrival rate defined per tier. This is due to the FE tier having to convey all traffic to avoid bottlenecks at this tier. However, SL and SDB tiers can be optimized to reduce overdimensioning. Then, we set the target arrival rate in the FE tier as in the overdimensioned scheme explained above:

(11) $$\lambda_{FE}={\bar{\lambda }}_{MBB}+\lambda_{mM2M}^{peak}+\lambda_{lM2M}^{intsmooth}$$

Since SLs are dimensioned differently for each traffic class, we set their target arrival rate differently. For MBB traffic, the mean arrival rate, for mM2M traffic, we apply moderate smoothing of traffic peaks, $\lambda_{mM2M}^{smoothpeak}$; and for lM2M, the traffic peak, $\lambda_{lM2M}^{peak}$.

Note that the mM2M pool of SL instances is not designed to support mM2M traffic peaks, but this is not expected to be a major issue as mM2M devices are assumed to be delay tolerant. Furthermore, as mentioned in Section 3, the SL tier is assumed to have enough memory resources to store all packets while they are queued. Then, we set the target arrival rate for each traffic class as:

(12) $$\begin{array}{cc} MBB\; & \lambda_{SL}={\bar{\lambda }}_{MBB}\\ mM2M\; & \lambda_{SL}=\lambda_{mM2M}^{smoothpeak}\\ lM2M\; & \lambda_{SL}=\lambda_{lM2M}^{peak} \end{array}$$

At the SDB tier, the target arrival rate is a sum of the target arrival rate of each class of SLs, then:

(13) $$\lambda_{SDB}={\bar{\lambda }}_{MBB}+\lambda_{mM2M}^{smoothpeak}+\lambda_{lM2M}^{peak}$$

#### 5.3.2. Traffic Shaper Scheme

**Figure 8:**
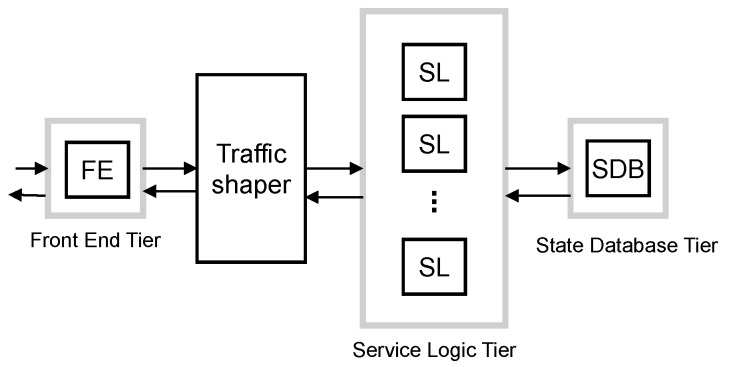
Traffic separated scheme block diagram.

The second proposed scheme adds a traffic shaper after the front end tier to control the traffic of each class to be processed by the SL and SDB tiers. This can be considered as a middle approach between the overdimensioned and the traffic separated schemes; see Figure 8.

As in the overdimensioned scheme, in SS, all resources are shared among all traffic classes. However, the traffic shaper can smooth traffic peaks and benefit some traffic classes through their shaping criteria. In addition, this scheme avoids the multiplexing loss of processing resources suffered by the separated traffic scheme due to the spare capacity of the dedicated workers that cannot be used for other traffics classes.

As in the overdimensioned scheme, each tier has to satisfy ${\bar{D}}_{j}$ with a target arrival rate. However, for this scheme, the SL and the SDB tiers have the same target arrival rate as they process the same traffic after the shaping of the traffic shaper. The target arrival rate used to design the FE tier is the same as in the traffic separated scheme. Therefore, it can be calculated as:

(14) $$\lambda_{FE}={\bar{\lambda }}_{MBB}+\lambda_{mM2M}^{peak}+\lambda_{lM2M}^{intsmooth}$$

We assume that the traffic shaper is implemented with a token bucket for each traffic class. The token rates of the buckets are set equal to the target arrival rate for each class, respectively. We set the target arrival rate for each class as in the traffic separated scheme. That is, for MBB traffic, the mean arrival rate; for mM2M traffic, we apply moderate smoothing of traffic peaks, $\lambda_{mM2M}^{smoothpeak}$; and for lM2M, the traffic peak, $\lambda_{lM2M}^{peak}$.

After the traffic shaper, the SL and the SDB tiers have a target arrival rate equal to the sum of the target arrival rate of all classes. Then, the target arrival rate can be calculated as:

(15) $$\lambda_{SL}=\lambda_{SDB}={\bar{\lambda }}_{MBB}+\lambda_{mM2M}^{smoothpeak}+\lambda_{lM2M}^{peak}$$

This design also requires enough memory resources at traffic shaper queues to store all packets while they are queued, as assumed in Section 3.

Table 2 summarizes the target arrival rate criteria of each baseline and proposed scheme.

## 6. Dimensioning

The dimensioning of the schemes presented above requires a model that provides an estimation of the service response time as a function of the processing resources. To do that, we assume the vMME model proposed in [32], which is based on the model of a typical cloud processing chain [35].

**Figure 9:**
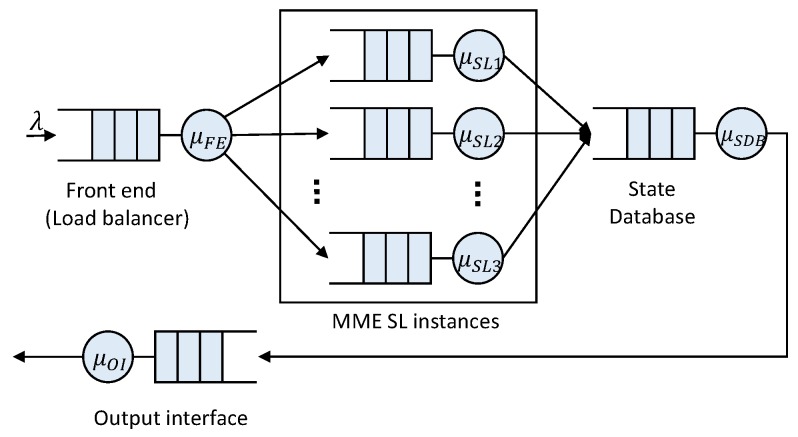
Queue model of the vMME.

This proposal uses Jackson’s open queuing network (see Figure 9) to model the vMME architecture (explained in Section 3). In this approach, the SDB and the FE tiers are modeled by M/M/1 queues, and the SL tier is modeled by an M/M/m queue.

Jackson’s theorem states that the numbers of messages in the system’s queues are independent of the other queues, and consequently, the service response time of the complete system is equal to the sum of the service response time of the queue in each tier. In the present work, we also assume that the SDB and the FE can be replicated, and therefore, we also model SDB and FE tiers by an M/M/m queue.

Given a target arrival rate, the goal of our vMME dimensioning is to determine the minimum number of instances required at each tier *j* to guarantee the mean response time budget ${\bar{D}}_{j}$. Since we consider Jackson’s open queuing network, the mean response time of the system $\bar{T}$ can be computed as:

(16) $$\bar{T}=\sum_{j}{\bar{T}}_{j}$$

where ${\bar{T}}_{j}$ is the mean response time at each tier *j* ∈{FE,SL,SDB}. Since we assume that each tier is modeled by an M/M/m queue, it holds that:

(17) $${\bar{T}}_{j}=\frac{1}{\mu_{j}}+\frac{C(m_{j},\rho_{j})}{m_{j}\cdot \mu_{j}-\lambda_{j}}$$

where $\rho_{j}=\frac{\lambda_{j}}{\mu_{j}}$, $\mu_{j}$ is the service rate of one tier instance, $\lambda_{j}$ is the target arrival rate considered for dimensioning at tier *j* (see Table 2), $m_{j}$ is the number of instances of the tier and $C(m_{j},\rho_{j})$ is Erlang’s C formula. $C(m_{j},\rho_{j})$ represents the probability that an arriving packet has to wait in the queue of the tier because all of the instances are busy, and it has the following expression:

(18) $$C\left(m_{j},\rho_{j}\right)=\frac{\left(\frac{{\left(m_{j}\cdot \rho_{j}\right)}^{m_{j}}}{m_{j}!}\right)\cdot \left(\frac{1}{1-\rho_{j}}\right)}{\sum_{k=0}^{m_{j}-1}\frac{{\left(m_{j}\cdot \rho_{j}\right)}^{k}}{k!}+\left(\frac{{\left(m_{j}\cdot \rho_{j}\right)}^{m_{j}}}{m_{j}!}\right)\cdot \left(\frac{1}{1-\rho_{j}}\right)}$$

The processing times of the FE, SDB and output interface are constant. However, the processing time of an SL is different for each control message [32]. Consequently, the mean service time of an SL, ${\bar{t}}_{SL}=\frac{1}{\mu_{SL}}$, will depend on the frequency with which each type of control procedure occurs. For this reason, ${\bar{t}}_{SL}$ will be different for each scheme considered in this work.

Let $t_{SRi}$, $t_{SRRi}$, $t_{HOi}$ and $t_{MO-CPCEOi}$ denote the processing time of the *i*-th message of the SR, SRR, HO and MO-CPCEO procedure, respectively. The mean service times of an SL for each scheme considered are summarized in Table 3.

We perform dimensioning for each tier individually. The dimensioning problem for each tier can be formulated as:

(19) $$m_{j}=\min\left\{M_{j}:{\bar{T}}_{j}\left(\lambda_{j},M_{j}\right)\leq {\bar{D}}_{j},M_{j}\in N\right\}$$

where ${\bar{D}}_{j}$ is the target mean response time for each tier. Hence, $m_{j}$ can be computed with a simple iterative algorithm that increases the number of tier instances until the condition ${\bar{T}}_{j}\left(\lambda_{j},M_{j}\right)\leq {\bar{D}}_{j}$ is met.

## 7. Evaluation

This section includes the simulation results obtained to evaluate the four vMME schemes (Section 5). After the simulation setup subsection, we compare the required resources and their associated costs in the dimensioning subsection. Later, we compare the delay experienced by each traffic class in the vMME with the four virtualized schemes.

### 7.1. Simulation Setup

Our evaluation methodology includes three steps, namely:

1. The dimensioning of each tier of the vMME model and the estimation of the associated cost using the target arrival rate as input; this estimation is done for a range of UEs and a given ratio of M2M devices per UE;
2. The generation of signaling traces for each traffic class (MBB, mM2M and lM2M); similarly, this trace generation is done assuming a specific number of UEs and a given ratio of M2M devices per UE;
3. Finally, the simulation of the vMME queuing model using the signaling trace as input.

We use the MATLAB Simulink framework to simulate the queuing system presented in Section 6, which provides the vMME response time experienced by a control plane message. The queuing model is fed with the traces of signaling packets generated by each traffic class. In the model, the service rates of the FE tier, SL tier, SDB tier and output interface are extracted from [32].

For the MO-CPCEO procedure, we obtained that the processing time needed at an SL instance is 145.05 μs. Other main parameters of the simulation are summarized in Table 4. To avoid excessively long simulations, we assume a period of time between mM2M events equal to 60 s.

To estimate the system running cost, we consider the Amazon EC2 service, with the costs and configuration detailed in Table 5. We assume a medium-sized CPU instance *m3.xlarge* with an average of $11.38\times 10^{9}$ float operations per second [36]. We use the price of the load balancing service provided by Amazon to estimate the cost of the FE tier. Our setup also includes the Amazon Aurora database [37], which is reported to provide $10^{5}$ updates/s transactions. The overall cost includes the per instance cost, the time-based rental fee and the data traffic processed.

### 7.2. Results

#### 7.2.1. Dimensioning

**Figure 10:**
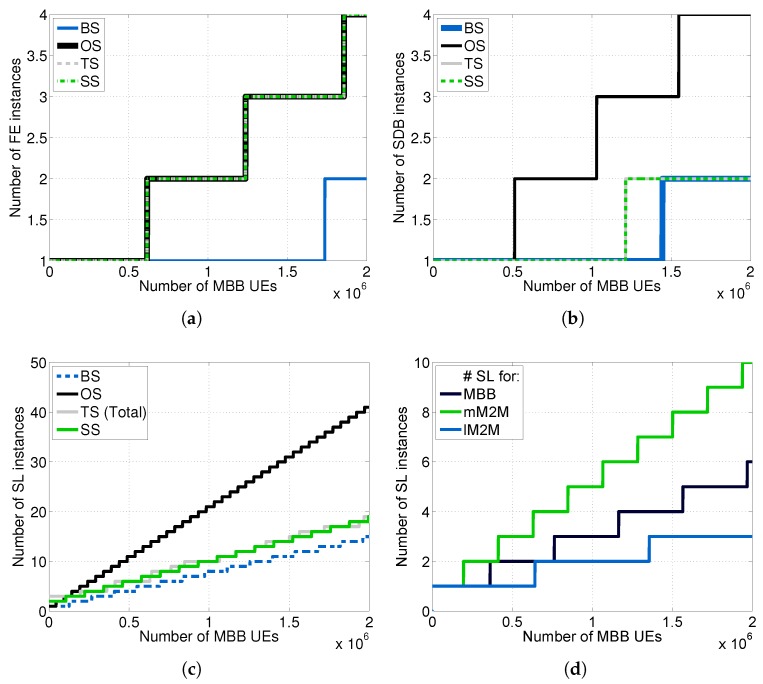
Dimensioning at each tier of the vMME model (ten M2M devices per MBB UE). (**a**) FE dimensioning; (**b**) SDB dimensioning; (**c**) SL dimensioning; (**d**) Detailed SL TS dimensioning.

We carried out the dimensioning at each tier of the vMME versus $N_{MBB}$ for all of the schemes considered (see Figure 10) by using the theoretical framework described in Section 6 and assuming ${\bar{D}}_{j}=1\;ms\;\forall \;j$.

**Figure 11:**
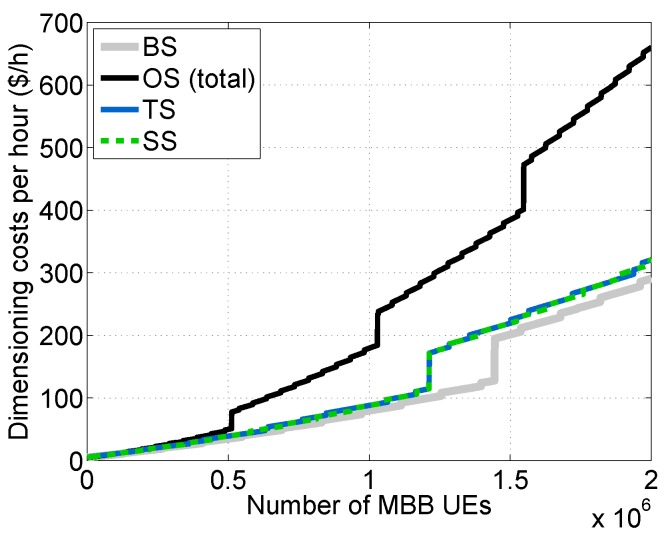
Dimensioning costs comparison per evaluated scheme.

Notably, to simplify the comparisons in the Traffic separated Scheme (TS), we set the same response time budget for MBB and lM2M traffic classes; however, this scheme enables one to set different budgets for each class. Note additionally that, for the TS scheme, the required number of SL instances $m_{SL}$ is the sum of the required number of SL instances for each type of traffic, which are depicted in Figure 10d. Additionally, we computed the cost per hour for each scheme considered (see Figure 11).

As was expected, the Overdimensioned Scheme (OS) demands the greatest amount of resources, being the most expensive scheme. Conversely, the Baseline Scheme (BS) is the least expensive one. The Traffic separated Scheme (TS) and the traffic Shaper Scheme (SS), which have a similar cost, achieve a noticeable reduction in cost in comparison with OS. This is mainly thanks to the isolation between traffic types in the TS case and the limitation imposed by the traffic shaper on the mM2M traffic arrival rate in the SS case. In such cases, the dimensioning at the SL and SDB tiers can be performed without considering $\lambda_{mM2M}^{peak}$, which is around 12.47-times greater than $\lambda_{lM2M}^{peak}$ in our experimental setup. However, both the TS and SS schemes are designed to satisfy the delay constraint for lM2M traffic.

Additionally, Table 6 summarizes the estimation of the memory consumption for each tier of the vMME. It assumes the same number of MBB UEs and M2M devices than the simulation setup. We use the number of SL instances from Figure 10c. We utilize 16,545 packets queued in the system. This number is the worst case of packets queued of the traffic separated scheme proposed, calculated from the results obtained in the next subsection.

#### 7.2.2. Delay

**Figure 12:**
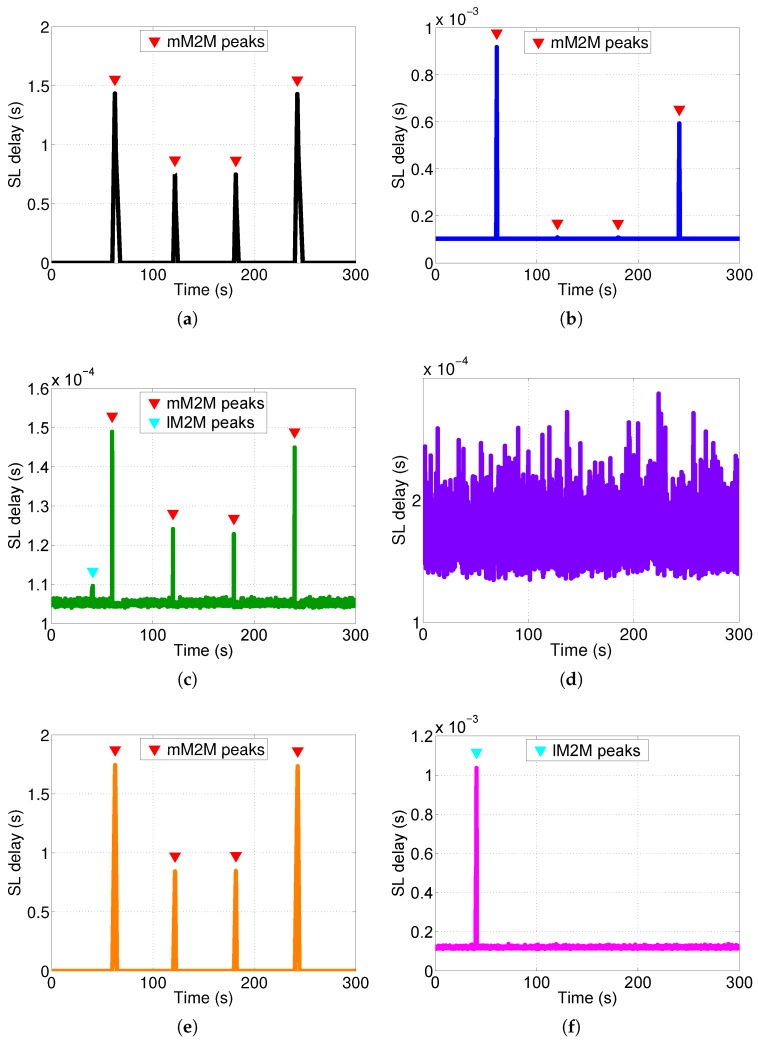
SLs’ filtered processing time for each scheme (ten M2M devices per MBB UE). (**a**) Baseline scheme; (**b**) Overdimensioned scheme; (**c**) Traffic shaper scheme; (**d**) Traffic separated scheme: MBB class; (**e**) Traffic separated scheme: mM2M class; (**f**) Traffic separated scheme: lM2M class.

**Figure 13:**
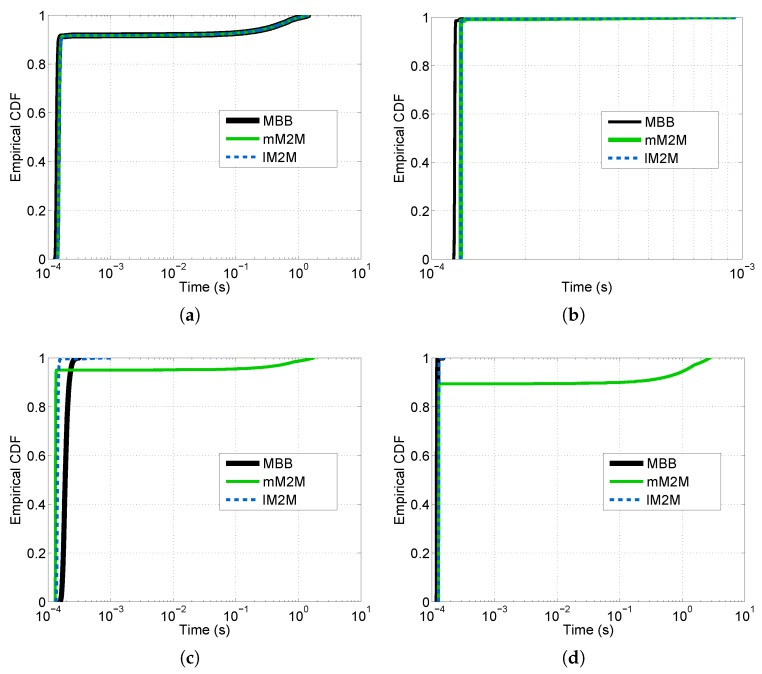
CDF of the filtered vMME delay for each scheme (ten M2M devices per MBB UE). (**a**) Baseline scheme; (**b**) Overdimensioned scheme; (**c**) Traffic separated scheme; (**d**) Traffic shaper scheme.

We studied the response time experienced by a control packet at the SL’s tier of the vMME for all of the schemes (see Figure 12). Additionally, we computed the CDF of the overall system response time, which is the sum of the delay experienced by a packet at each tier of the vMME (see Figure 13). To that end, we generated a signaling trace for 636,000 MBB UEs and 300 s of duration. The trace includes the three considered classes. Table 7 summarizes the results for this point. The number of UEs is selected such that the processing capacity of the OS scheme and the lM2M SL pool in the TS scheme are about to require an additional SL instance to satisfy ${\bar{D}}_{SL}$. However, with this number of users, the remaining schemes do not experience the same situation. The same signaling traces were used for the four schemes considered. The response time results are filtered with a simple 90-ms moving average. The results show that response time is higher than the target mean response time at the SL tier (${\bar{D}}_{SL}$ = 1 ms) during the mM2M alarm events for the BS case (Figure 12a). That is because the SL tier is under-dimensioned to support the mM2M traffic peaks. Consequently, in such situations, the mM2M traffic might delay the other traffic types, which may be delay-sensitive, such as lM2M (see Figure 13a). On the contrary, for the OS case, the response time at the SL tier is all of the time below ${\bar{D}}_{SL}$ since the system is overdimensioned (Figure 12b).

For the SS approach, the SL tier response time always meets the condition ${\bar{T}}_{SL}\leq {\bar{D}}_{SL}$ (Figure 12c). That is because the mM2M traffic peaks are limited by the traffic shaper. Moreover, during the lM2M traffic peaks, the system takes advantage of the multiplexing gain. In the TS case, the lM2M pool of the SL tier also meets the condition ${\bar{T}}_{SL}\leq {\bar{D}}_{SL}$ during the lM2M traffic peak (see Figure 12f). On the contrary, the mM2M pool of the SL tier exceeds by several orders of magnitude the response time budget during and after the mM2M traffic peaks (see Figure 12e), as moderate smoothing of the peaks is applied. The MBB pool of the SL tier also meets the response time budget condition (see Figure 12d). Recall that, with the selected number of UEs, only the OS (Figure 12b) and the lM2M class in the TS scheme (Figure 12f) have a processing load close to the dimensioned capacity.

## 8. Conclusions

In this paper, we propose two designs for a virtualized MME, which aim at facilitating IoT support in 5G systems. The first proposed design partially separates the processing resources devoted to each traffic class; while the second design includes traffic shaping to control the traffic of each class.

We have considered three traffic classes: MBB, massive M2M and low latency M2M. In M2M communications, we have included M2M events to analyze the performance of the virtualized MME. M2M events considered are caused by the report synchronization of massive M2M devices and alarm events of low latency M2M devices. Additionally, we assume the use of the CPCEO procedure to transfer these reports from M2M devices.

We have compared our proposed designs with two other schemes: (i) a baseline virtualized MME design, which does not apply such resource separation; (ii) an overdimensioned virtualized MME.

The reported comparisons include: (i) dimensioning of the required resources; (ii) estimation of the costs based on the model of Amazon EC2; (iii) the evaluation of the response time of the virtualized MME schemes for each traffic class.

After the conducted simulations, the results show that our proposed schemes provide much lower costs than the overdimensioned scheme while they satisfy the exigent delay requirements of MBB and low latency M2M communications. Furthermore, the comparison of the traffic separation scheme and the traffic shaper scheme shows that the multiplexing gain of the latter provides benefits in terms of latency reduction. However, the traffic separation scheme enables one to have different delay requirements for each traffic class, and additionally, it can isolate their performance.

In addition to the above results, we identify the following advantages of the considered solution: (i) CPCEO procedures mitigate the signaling explosion generated by a huge number of M2M connected devices. However, it increases the processing load on the control plane of the EPC. (ii) NFV increases the scalability of the network to deal with such load increase of signaling. (iii) The usage of our proposed schemes further optimize the costs while satisfying the delay demands.

However, our solution has also the following implications: (i) the dimensioning of the resources is more complex; (ii) CPCEO procedures use the transport network of the control plane to send data packets, which imposes an additional load. Furthermore, regarding the bottlenecks of the proposed schemes, the state database tier is critical. This is due to the state database scaling with certain constraints, and our solution makes heavy use of it. Moreover, the service logic tier design is determinant, as it considerably affects the performance of each traffic class.

## Figures and Tables

**Table 1:** Primary definitions. FE, Front End; SL, Service Logic; SDB, State Database; MBB, Mobile Broadband; UE, User Equipment; mM2M, massive M2M; MO-CPCEO, Mobile-Originated Control Plane Cellular IoT Evolved packet system Optimization.

Notation	Description
${\bar{T}}_{j}$	Mean response time for each tier (where $j\in \left\{FE,SL,SDB\right\}$)
${\bar{D}}_{j}$	Target mean response time for tier *j*
$N_{MBB}$	Number of MBB UEs
$N_{h}$	Number of each class of M2M devices (where $h\in \left\{mM2M,lM2M\right\}$)
${\bar{\lambda }}_{SR}$	Mean generation rate per MBB UE of *Service Request*
${\bar{\lambda }}_{SRR}$	Mean generation rate per MBB UE of *Service Release*
${\bar{\lambda }}_{HO}$	Mean generation rate per MBB UE of *Handover*
${\bar{\lambda }}_{MBB}$	Mean arrival rate of control messages generated by MBB UEs
${\bar{\lambda }}_{active}$	Mean M2M device packet rate in the *active* state
${\bar{\lambda }}_{alarm}$	Mean M2M device packet rate in the *alarm* state
$a_{h}$	Percent of M2M devices of each class that changes to the *alarm* state in an M2M event
$p_{h}$	Percent of each class of M2M devices
$p_{h}^{e}$	Percent in a certain instant of time an M2M device is in the *alarm* state
*r*	Ratio of M2M devices per MBB UE
${\bar{\lambda }}_{h}^{v}$	Mean arrival rate of *MO-CPCEO* per M2M class in a observation period (where $v\in \left\{active,event,maxevent\right\}$)
${\bar{\lambda }}_{h}$	Mean arrival rate of *MO-CPCEO* control messages generated per M2M class
$w_{h}$	Weighting factor of arrival rates for the absence or not of M2M events per M2M class
$\lambda_{h}^{t}$	Target arrival rate for dimensioning
$\lambda_{h}^{intsmooth}$	Target arrival rate with intense smoothing of peaks
$\lambda_{h}^{smoothpeak}$	Target arrival rate with moderate smoothing of peaks
$\lambda_{h}^{peak}$	Target arrival rate of peak traffic
$\lambda_{FE}$	Front end target arrival rate
$\lambda_{SL}$	Service logic target arrival rate
$\lambda_{SDB}$	State database target arrival rate
$\mu_{j}$	Service rate of tier instance *j*
$m_{j}$	Number of instances of tier *j*

**Table 2:** Target arrival rate at each tier for the proposed schemes.

	Tier	Front End (FE)	Service Logic (SL)		State Database (SDB)
Scheme					
Baseline Scheme (BS)		$\lambda_{FE}={\bar{\lambda }}_{MBB}+\lambda_{mM2M}^{intsmooth}+\lambda_{lM2M}^{intsmooth}$	$\lambda_{SL}=\lambda_{FE}$		$\lambda_{SDB}=\lambda_{SL}$
Overdimensioned Scheme (OS)		$\lambda_{FE}={\bar{\lambda }}_{MBB}+\lambda_{mM2M}^{peak}+\lambda_{lM2M}^{intsmooth}$	$\lambda_{SL}=\lambda_{FE}$		$\lambda_{SDB}=\lambda_{SL}$
Traffic separated Scheme (TS)		$\lambda_{FE}={\bar{\lambda }}_{MBB}+\lambda_{mM2M}^{peak}+\lambda_{lM2M}^{intsmooth}$	MBB	$\lambda_{SL}={\bar{\lambda }}_{MBB}$	$\lambda_{SDB}={\bar{\lambda }}_{MBB}+\lambda_{mM2M}^{smoothpeak}+\lambda_{lM2M}^{peak}$
			mM2M	$\lambda_{SL}=\lambda_{mM2M}^{smoothpeak}$	
			lM2M	$\lambda_{SL}=\lambda_{lM2M}^{peak}$	
Traffic Shaper Scheme (SS)		$\lambda_{FE}={\bar{\lambda }}_{MBB}+\lambda_{mM2M}^{peak}+\lambda_{lM2M}^{intsmooth}$	$\lambda_{SL}={\bar{\lambda }}_{MBB}+\lambda_{mM2M}^{smoothpeak}+\lambda_{lM2M}^{peak}$		$\lambda_{SDB}=\lambda_{SL}$

**Table 3:** SL’s mean service time.

Scheme	Mean Service Time	
Baseline Scheme (BS)	${\bar{t}}_{SL}=\frac{N_{MBB}\cdot \left({\bar{\lambda }}_{SR}\cdot \left(t_{SR1}+t_{SR2}+t_{SR3}\right)+{\bar{\lambda }}_{SRR}\cdot \left(t_{SR1}+t_{SR2}+t_{SR3}\right)+{\bar{\lambda }}_{HO}\cdot \left(t_{HO1}+t_{HO2}\right)\right)+\left(\lambda_{mM2M}^{intsmooth}+\lambda_{lM2M}^{intsmooth}\right)\cdot t_{MO-CPCEO1}}{{\bar{\lambda }}_{MBB}+\lambda_{mM2M}^{intsmooth}+\lambda_{lM2M}^{intsmooth}}$	
Overdimensioned Scheme (OS)	${\bar{t}}_{SL}=\frac{N_{MBB}\cdot \left({\bar{\lambda }}_{SR}\cdot \left(t_{SR1}+t_{SR2}+t_{SR3}\right)+{\bar{\lambda }}_{SRR}\cdot \left(t_{SR1}+t_{SR2}+t_{SR3}\right)+{\bar{\lambda }}_{HO}\cdot \left(t_{HO1}+t_{HO2}\right)\right)+\left(\lambda_{mM2M}^{peak}+\lambda_{lM2M}^{intsmooth}\right)\cdot t_{MO-CPCEO1}}{{\bar{\lambda }}_{MBB}+\lambda_{mM2M}^{peak}+\lambda_{lM2M}^{intsmooth}}$	
Traffic separated Scheme (TS)	MBB	${\bar{t}}_{SL}=\frac{N_{MBB}\cdot \left({\bar{\lambda }}_{SR}\cdot \left(t_{SR1}+t_{SR2}+t_{SR3}\right)+{\bar{\lambda }}_{SRR}\cdot \left(t_{SR1}+t_{SR2}+t_{SR3}\right)+{\bar{\lambda }}_{HO}\cdot \left(t_{HO1}+t_{HO2}\right)\right)}{{\bar{\lambda }}_{MBB}}$
	mM2M	${\bar{t}}_{SL}=t_{MO-CPCEO1}$
	lM2M	${\bar{t}}_{SL}=t_{MO-CPCEO1}$
Traffic Shaper Scheme (SS)	${\bar{t}}_{SL}=\frac{N_{MBB}\cdot \left({\bar{\lambda }}_{SR}\cdot \left(t_{SR1}+t_{SR2}+t_{SR3}\right)+{\bar{\lambda }}_{SRR}\cdot \left(t_{SR1}+t_{SR2}+t_{SR3}\right)+{\bar{\lambda }}_{HO}\cdot \left(t_{HO1}+t_{HO2}\right)\right)+\left(\lambda_{mM2M}^{smoothpeak}+\lambda_{lM2M}^{peak}\right)\cdot t_{MO-CPCEO1}}{{\bar{\lambda }}_{MBB}+\lambda_{mM2M}^{smoothpeak}+\lambda_{lM2M}^{peak}}$	

**Table 4:** Parameters’ configuration.

Simulation Parameters		
Simulation Time		300 s
MBB UEs		636,000
M2M devices per MBB UE		10
M2M packet size		200 B [19]
M2M event duration		1 s
FE mean response time budget		1 ms
SL mean response time budget		1 ms
SDB mean response time budget		1 ms
Unweighted sliding-average smooth		90 ms
$w_{mM2M}$		0.1
$w_{lM2M}$		0.0033
**Traffic Models**		
Mobile Broadband (MBB)	${\bar{\lambda }}_{SR}$	0.0045 pkt /s [32]
	${\bar{\lambda }}_{SRR}$	0.0045 pkt/s [32]
	${\bar{\lambda }}_{HO}$	0.0012 pkt/s [32]
Both M2M devices	${\bar{\lambda }}_{active}$	0.0033 pkt/s
	${\bar{\lambda }}_{alarm}$	0.033 pkt/s
Massive M2M (mM2M)	Percentage of mM2M devices	90%
	Event’s period	60 s
	Event’s magnitude values	[10, 30, 50, 8] %
	Event magnitude values’ probability mass	[5, 60, 20, 15] %
Low latency M2M (lM2M)	Percentage of lM2M devices	10%
	Event’s period	Unique (at 40 s of the simulation)
	Event’s magnitude value	33%

**Table 5:** Cloud service configuration and cost calculation.

Cost	Configuration	Calculation	
$C_{ci_{type}}\left(k\right)$	*m3.xlarge* instance rental (0.266 $/h)	0.266/3600	
$C_{ci_{stor}}\left(k\right)$	Local storage (10 GB/month) and optimized data access (0.025 $/h).	10·0.10+0.025/3600	
$C_{ci_{thro}}\left(k\right)$	Data sent from the data center, (λ (message/s)·200 (byte/message))	0.000 ($)/GB	First GB/month
		0.090 ($)/GB	Up to 10 TB/month
		0.085 ($)/GB	Next 40 TB/month
		0.070 ($)/GB	Next 100 TB/month
		0.050 ($)/GB	Next 350 TB/month
$C_{db_{type}}\left(k\right)$	Aurora db.r3.8xlarge instance (4.64 $/h)	4.64/3600	
$C_{db_{stor}}\left(k\right)$	0.1 $ per GB/month, for a total database size of $N_{U}\cdot 1$ KB	$(0.1\cdot N_{U}\cdot 1024\cdot \lambda /10^{9})/$2,628,000	
$C_{db_{thro}}\left(k\right)$	0.2 $ per million transactions/month	$0.2\cdot \lambda /10^{6}$	
$C_{b_{type}}\left(k\right)$	Service fee of 0.025 $/month	0.025/2,628,000	
$C_{b_{thro}}\left(k\right)$	0.008 $ per GB serviced, supposing $O_{size}=200$ bytes	$\lambda \cdot 0.008\cdot 200/10^{9}$	

**Table 6:** Memory consumption estimation (UE context extracted from [38,39]).

Element	Memory Consumption	Sample Scenario $(N_{\mathrm{MBB}}=636,000$ MBB UEs $N_{M2M}=10\cdot N_{\mathrm{MBB}})$
State database	UE context: 264 B/UE	264 B/UE ·$\left(N_{MBB}+N_{M2M}\right)$ = 1846 MB
Service logic	Operating System ROM: 1000 MB/instance Operating System RAM: 400 MB/instance [40] UE context: 264 B/UE Packet size: 200 B	ROM: 1000 MB · 7 instances = 7000 MB RAM: ( 400 MB + 264 B/UE ) · 7 instances + 16,545 packets · 200 B/packet = 2803 MB

**Table 7:** vMME model dimensioning at the simulation point.

	Tier	Number of FE Instances	Number of SL Instances		Number of SDB Instances
Scheme					
BS		1	5		1
OS		2	13		2
TS		2	MBB	2	1
			mM2M	4	
			lM2M	1	
SS		2	7		1
